# A genome assembly of the California poppy, *Eschscholzia californica*

**DOI:** 10.1093/jhered/esaf058

**Published:** 2025-08-28

**Authors:** Peter T Nguyen, Merly Escalona, Elizabeth Ryan, Courtney Miller, Mohan P A Marimuthu, Colin W Fairbairn, William Seligmann, Noravit Chumchim, Oanh Nguyen, Erin Toffelmier, H Bradley Shaffer, Jason P Sexton, Elsa E Cleland

**Affiliations:** Department of Life and Environmental Sciences, University of California, Merced, CA, United States; Department of Biomolecular Engineering, University of California, Santa Cruz, CA, United States; Department of Ecology, Behavior & Evolution, University of California, San Diego, CA, United States; Department of Ecology and Evolutionary Biology, University of California, Los Angeles, CA, United States; DNA Technologies and Expression Analysis Core Laboratory, Genome Center, University of California, Davis, CA, United States; Department of Ecology and Evolutionary Biology, University of California, Santa Cruz, CA 95064, United States; Department of Ecology and Evolutionary Biology, University of California, Santa Cruz, CA 95064, United States; DNA Technologies and Expression Analysis Core Laboratory, Genome Center, University of California, Davis, CA, United States; DNA Technologies and Expression Analysis Core Laboratory, Genome Center, University of California, Davis, CA, United States; Department of Ecology and Evolutionary Biology, University of California, Los Angeles, CA, United States; La Kretz Center for California Conservation Science, Institute of the Environment and Sustainability, University of California, Los Angeles, CA, United States; Department of Ecology and Evolutionary Biology, University of California, Los Angeles, CA, United States; La Kretz Center for California Conservation Science, Institute of the Environment and Sustainability, University of California, Los Angeles, CA, United States; Department of Life and Environmental Sciences, University of California, Merced, CA, United States; Department of Ecology, Behavior & Evolution, University of California, San Diego, CA, United States

**Keywords:** California Conservation Genomics Project (CCGP), conservation, genomics, restoration, plant adaptation

## Abstract

The California poppy (*Eschscholzia californica*), a native wildflower of western North America and the state wildflower of California, is characterized by extensive ecological variation and adaptation to diverse climatic conditions. Its broad geographic range and adaptability make it a valuable model for studying how plants respond to changing environmental conditions. Here, we present an updated, near-chromosome-level genome assembly for *E. californica*, developed as part of the California Conservation Genomics Project. This assembly spans 0.401 Gb and represents an advancement over previous versions, with a scaffold N50 of 66.4 Mb, a contig N50 of 11.8 Mb, and BUSCO completeness of 99.2%, providing near-complete genomic coverage. The enhanced genome assembly described here facilitates precise whole-genome resequencing, providing insights into genetic diversity and gene flow between populations—key factors in understanding the adaptive mechanisms that will support the species’ survival in the face of environmental challenges.

## Introduction

The California poppy (*Eschscholzia californica*), native to western North America, is characterized by distinctive orange, cup-shaped flowers, broad ecological tolerances, and seasonal floral displays. It has become naturalized or invasive in many parts of the world. *Eschscholzia californica* is known for producing large-scale floral events, commonly referred to as ‘super blooms,’ during periods of high rainfall. As California’s state flower, it is used in horticulture, gardening, habitat restoration, and ecological restoration efforts (Smith, 2010). *Eschscholzia californica* holds cultural and medicinal significance among Indigenous communities in California, where it has been traditionally used for various purposes, including as a sedative and analgesic (Anderson 2007). *Eschscholzia californica* has a long history as a model in evolutionary developmental biology, particularly for studies of flower and fruit development, and it remains highly valued as an evo-devo system today (Pabón-Mora et al., 2012; Becker et al., 2023). In more arid parts of its range, *E. californica* adopts an annual life cycle, whereas in mesic environments it persists as a short-lived perennial, having a larger taproot that facilitates deep resource acquisition and storage (Still et al. 2023). The species thrives across a broad geographical range, from southern Oregon to northern Baja California, Mexico, and at elevations from sea level to 2500 meters, where it often coexists with diverse wildflower communities and long-lived woody species, including native oaks. Its wide distribution and adaptability make *E. californica* an informative model for studying natural selection across environmental gradients. Previous research has revealed significant phenotypic diversity throughout its range, including clinal variations in traits shaped by historical natural selection in response to climate (Cook 1962; Leger and Rice 2003).

The species’ widespread distribution and significant genetic variation provide opportunities for investigating the genetic mechanisms underlying drought adaptation (Ryan and Cleland 2021). Across its range, populations exhibit substantial variation in traits associated with water use and drought escape, including flowering time, leaf morphology, and growth rate (Ryan and Cleland 2021). Previous studies have documented clinal trait variation consistent with local adaptation to aridity, but the underlying genetic mechanisms remain largely unresolved (Leger and Rice, 2007). For example, populations of *E. californica* in Chile exhibit increased size and fecundity compared to native populations in California, suggesting a rapid response to selection for traits that promote fast growth and development in the introduced range (Leger and Rice 2003). A high-quality reference genome provides the foundation for linking these ecophysiological traits to specific genomic regions under selection. For example, with population resequencing data, it is possible to link specific single nucleotide polymorphism (SNPs) to drought-related traits, test for selection in arid versus mesic populations, and evaluate how gene flow shapes the distribution of adaptive variants across the landscape (Lotterhos and Whitlock 2015). The newly assembled genome now enables genome-wide association studies and genotype–environment association analyses to investigate the genetic basis of drought adaptation in *E. californica*, building on approaches shown to be effective in other wild species (Santure and Garant 2018; Chambers et al., 2023). This type of molecular approach is especially useful for conservation and restoration planning, where identifying locally adapted genotypes can inform seed sourcing and landscape-level management under future climate scenarios (Funk et al., 2019). In this vein, a reference genome can help evaluate whether seed sources are genetically suited to local environmental conditions, enhancing restoration success (USDA NRCS, n.d.). Altogether, the genome provides a tool to connect ecological and evolutionary patterns with practical restoration work, assisting efforts on the ground with the potential to improve understanding of local adaptation and genetic diversity in *E. californica*.

To support these goals, we present a near-chromosome-level genome assembly for *E.californica*, developed as part of the California Conservation Genomics Project (CCGP). The primary goal of the CCGP is to improve understanding of genomic variation across California through the comprehensive sequencing of approximately 150 species, many of which are of conservation concern (Fiedler et al. 2022; Shaffer et al. 2022). Although *E. californica* is not endangered, its broad distribution, ecological breadth, and widespread use in restoration projects make it an ideal system for both basic and applied research (Vasey and Holl 2007; Barry et al., 2006). In concert with assessing plant phenotypes, genomic data can inform restoration by identifying hearty genotypes, improving seed sourcing, and enabling long-term monitoring of genetic variation that may influence adaptive potential (Breed et al. 2019). This is especially true for *E. californica*, as it relies on a diverse range of generalist pollinators, which can move freely across fragmented habitats (Cook 1962). Pollinators allow pollen, thus genes, to flow between otherwise isolated populations. By analyzing genomic variation across populations, we can uncover patterns of gene flow shaped by pollinator movement and landscape barriers, seeing how connectivity is maintained or disrupted (Kwak et al., 1998). Overall, understanding how gene flow shapes genomic variation across these environments can inform landscape-level management of genetic connectivity (Keller et al., 2015; Shaffer et al. 2022). With further sequencing of individuals and comparison to the reference, the *E. californica* genome provides the resolution needed to detect patterns of connectivity, identify loci under selection, and evaluating the role of gene flow in maintaining or constraining local adaptation (Shaffer et al. 2022; Chambers et al., 2023). This reference genome will provide a foundation for future studies on the ecological and evolutionary dynamics of *E. californica*, while also offering value for conservation genomics and climate-resilient restoration strategies.

## Methods

### Biological materials

Seeds for *E. californica* were sourced from a wild population at the McLaughlin Reserve, part of the University of California Natural Reserve System (38.8602°N, −122.4166°W, DOI: 10.21973/N3W08D). The seeds were grown in local topsoil in the University of California San Diego greenhouses. On 25 March 2021, fresh *E. californica* leaves (approximately 4 grams) were collected from a single *E. californica* individual for high molecular weight DNA extraction. Separate tissue samples from this same individual were used to prepare the HiFi and Omni-C sequencing libraries. The collected material was placed in sterile 5 mL cryovials (Cole-Palmer) and transported on dry ice to the UC Davis Genome Center and the UC Santa Cruz Paleogenomics Lab for processing. Upon arrival, the material was immediately stored at −80°C for long-term preservation.

### HiFi methods

We extracted high molecular weight (HMW) genomic DNA (gDNA) from 300 mg of leaves (Sample id: CP2) using the cetyltrimethylammonium bromide (CTAB) method as described in Inglis et al. 2018, with the following modifications: (i) we used sodium metabisulfite (1% w/v) instead of 2-mercaptoethanol (1% v/v) in the sorbitol wash buffer and CTAB solution; (ii) we repeated the tissue homogenate wash steps until the supernatant turned clear; (iii) we performed the CTAB lysis step at 45°C and (iv) the chloroform extraction step twice using ice-cold chloroform. Extracted HMW DNA was further purified using the high-salt-phenol-chloroform (Pacific BioSciences—PacBio, CA) protocol, DNA purity was estimated by absorbance ratios (260/280 = 1.81 and 260/230 = 2.02) measured using a NanoDrop ND-1000 spectrophotometer (Thermo Fisher Scientific, MA), DNA yield (3 μg) was quantified using a Quantus Fluorometer (QuantiFluor ONE dsDNA Dye assay; Promega, WI), and the size distribution of the DNA was estimated using the Femto Pulse system (Agilent, CA), where 60% of the DNA fragments were found to be 30 kilobases (kb) or longer.

The HiFi SMRTbell library was constructed using the SMRTbell prep kit 3.0 (PacBio, CA; Cat. #102-182-700) according to the manufacturer’s instructions. HMW gDNA was sheared to a target DNA size distribution between 15 and 18 kb using Diagenode’s Megaruptor 3 system (Diagenode, Belgium; cat. B06010003). The sheared gDNA was concentrated using 1X of SMRTbell cleanup beads provided in the SMRTbell prep kit 3.0 for the repair and a-tailing incubation at 37°C for 30 min and 65°C for 5 min, followed by ligation of overhang adapters at 20°C for 30 min, clean-up using 1X SMRTbell cleanup beads, and nuclease treatment at 37°C for 15 min. The SMRTbell library was size-selected using 3.1X of 35% v/v diluted AMPure PB beads (PacBio, Cat. #100–265-900) to progressively remove SMRTbell templates < 5 kb. The 15–18 kb average HiFi SMRTbell library was sequenced at UC Davis DNA Technologies Core (Davis, CA) using one 8 M SMRT cell (PacBio, Cat #101–389-001), Sequel IIe sequencing chemistry 2.0, and 30-h movies each on a PacBio Sequel IIe sequencer.

### Omni-C methods

The Omni-C library was prepared using the Dovetail™ Omni-C™ Kit (Dovetail Genomics, CA) according to the manufacturer’s protocol with slight modifications. First, specimen tissue (leaves, ID:CP5-McLa7) was thoroughly ground with a mortar and pestle while cooled with liquid nitrogen. Nuclear isolation was then performed using published methods (Workman et al. 2018). Subsequently, chromatin was fixed in place in the nucleus and digested under various conditions of DNase I until a suitable fragment length distribution of DNA molecules was obtained. Chromatin ends were repaired and ligated to a biotinylated bridge adapter followed by proximity ligation of adapter-containing ends. After proximity ligation, crosslinks were reversed and the DNA was purified from proteins. Purified DNA was treated to remove biotin that was not internal to ligated fragments. An NGS library was generated using an NEB Ultra II DNA Library Prep kit (New England Biolabs, MA) with an Illumina compatible y-adaptor. Biotin-containing fragments were then captured using streptavidin beads. The post capture product was split into two replicates prior to PCR enrichment to preserve library complexity with each replicate receiving unique dual indices. Sequencing was performed at the Center for Advanced Technologies at the University of California, San Francisco (UCSF CAT) on an Illumina NovaSeq X platform (Illumina, CA) to generate approximately 100 million 2 × 150 bp read pairs per GB genome size.

### Nuclear genome assembly

We assembled the genome of *E. californica* following the CCGP assembly pipeline, which uses PacBio HiFi reads and Omni-C data to produce high-quality and highly contiguous genome assemblies. The pipeline is outlined in Table 1 and lists the tools and non-default parameters used in the assembly process. First, we removed the remnants adapter sequences from the PacBio HiFi dataset using HiFiAdapterFilt (Sim et al. 2022) and generated the initial phased diploid assembly using HiFiasm (Cheng et al. 2021) on Hi-C mode, with the filtered PacBio HiFi reads and the Omni-C dataset. We aligned the Omni-C data to both assemblies following the Arima Genomics Mapping Pipeline (https://github.com/ArimaGenomics/mapping_pipeline) and then scaffolded both assemblies with SALSA (Ghurye et al. 2017; Ghurye et al. 2019).

**Table 1:** Assembly pipeline and software used.

Assembly step	Software and any non-default options	Version	Reference
**Initial assembly**			
**Filtering PacBio HiFi adapters**	HiFiAdapterFilt	Commit 64d1c7b	Sim et al. 2022
**K-mer counting**	Meryl (k = 21)	1	https://github.com/marbl/meryl
**Estimation of genome size and heterozygosity**	GenomeScope	2	Ranallo-Benavidez et al. 2020
***De novo assembly (contiging)***	HiFiasm (Hi-C Mode, −primary, output hic.hap1.p_ctg, hic.hap2.p_ctg)	0.19.5-r592	Cheng et al. 2021, Cheng et al. 2022
**Scaffolding**			
**Omni-C data alignment**	Arima Genomics Mapping Pipeline	Commit 2e74ea4	https://github.com/ArimaGenomics/mapping_pipeline
**Arima Genomics Mapping Pipeline (AGMP)**	BWA-MEM	0.7.17-r1188	Li 2013
	samtools	1.11	Danecek et al. 2021
	filter_five_end.pl (AGMP)	Commit 2e74ea4	https://github.com/ArimaGenomics/mapping_pipeline
	two_read_bam_combiner.pl (AGMP)	Commit 2e74ea4	https://github.com/ArimaGenomics/mapping_pipeline
	picard	2.27.5	https://broadinstitute.github.io/picard/
**Omni-C Scaffolding**	SALSA (-DNASE, −i 20, −p yes)	2	Ghurye et al. 2017, Ghurye et al. 2019
**Omni-C Contact map generation**			
**Short-read alignment**	BWA-MEM (-5SP)	0.7.17-r1188	Li 2013
**SAM/BAM processing**	samtools	1.11	Danecek et al. 2021
**SAM/BAM filtering**	pairtools	0.3.0	Open2C et al. 2024
**Pairs indexing**	pairix	0.3.7	Lee et al. 2022
**Matrix generation**	cooler	0.8.10	Abdennur and Mirny 2020
**Matrix balancing**	hicExplorer (hicCorrectmatrix correct --filterThreshold −2 4)	3.6	Ramírez et al. 2018
**Contact map visualization**	HiGlass	2.1.11	Kerpedjiev et al. 2018
	PretextMap	0.1.4	https://github.com/wtsi-hpag/PretextView
	PretextView	0.1.5	https://github.com/wtsi-hpag/PretextMap
	PretextSnapshot	0.0.3	https://github.com/wtsi-hpag/PretextSnapshot
**Manual curation tools**	Rapid curation pipeline (Wellcome Trust Sanger Institute, Genome Reference Informatics Team)	Commit 7acf220c	https://gitlab.com/wtsi-grit/rapid-curation
**Genome quality assessment**			
**Basic assembly metrics**	QUAST (−-est-ref-size)	5.0.2	Gurevich et al. 2013
**Assembly completeness**	BUSCO (−m geno, −l embryophyta)	5.0.0	Manni et al. 2021
	Merqury	2020 January 29	Rhie et al. 2020
**Contamination screening**			
**Local alignment tool**	BLAST+ (−db nt, −outfmt ‘6 qseqid staxids bitscore std’, −max_target_seqs 1, −max_hsps 1, −evalue 1e-25)	2.15	Camacho et al. 2009
**General contamination screening**	BlobToolKit (HiFi coverage, BUSCO = embryophyta, NCBI Taxa ID = 3467)	2.3.3	Challis et al. 2020
**Chloroplast assembly**			
**de novo genome assembly**	Oatk (syncasm -k 1001 -c 400)	1	https://github.com/c-zhou/oatk
**Sequence alignment**	lastz (−-nogapped,--notransition, −step = 20)	1.04.15	Harris 2017
**Alignment visualization**	LAJ (http://globin.cse.psu.edu/dist/laj/)	2005 December 14	Wilson et al. 2001
**Genome annotation**	GeSeq (https://chlorobox.mpimp-golm.mpg.de/geseq.html)	2021	Tillich et al. 2017

**Fig. 1:**
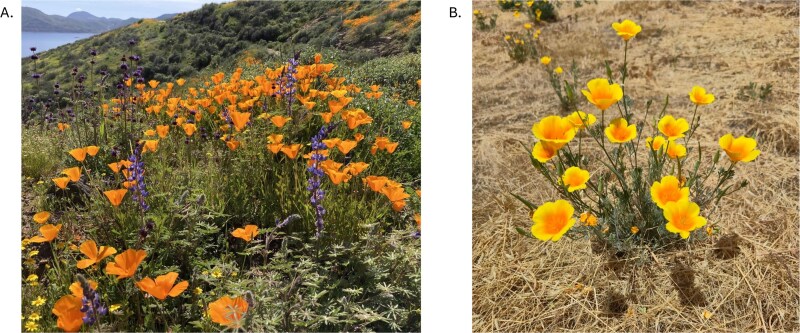
(A) Habitat view of *Eschscholzia californica* at Diamond Valley Lake, California, highlighting its natural growth conditions. (B) Detailed close-up of an individual *E. californica* plant at the University of California, Merced, illustrating the flower morphology and developing seed pods. Photo credits: (A) Elsa Cleland; (B) Hayden Nguyen.

Assemblies were manually curated by iteratively generating and analysing their corresponding Omni-C contact maps. To generate the contact maps, we aligned the Omni-C data with BWA-MEM (Li 2013), identified ligation junctions, and generated Omni-C pairs (Lee et al. 2022) using pairtools (Open2C et al. 2024). We generated a multi-resolution Omni-C matrix with cooler (Abdennur and Mirny 2020) and balanced it with hicExplorer (Ramírez et al. 2018). We used HiGlass (Kerpedjiev et al. 2018) and the PretextSuite (https://github.com/wtsi-hpag/PretextView; https://github.com/wtsi-hpag/PretextMap; https://github.com/wtsi-hpag/PretextSnapshot) to visualize the contact maps where we identified misassemblies and misjoins, and finally modified the assemblies using the Rapid Curation pipeline from the Wellcome Trust Sanger Institute, Genome Reference Informatics Team (https://gitlab.com/wtsi-grit/rapid-curation). Some of the remaining gaps (joins generated during scaffolding and/or curation) were closed using the PacBio HiFi reads and YAGCloser (https://github.com/merlyescalona/yagcloser). Finally, we checked for contamination using the BlobToolKit Framework (Challis et al. 2020).

### Genome quality assessment

We generated k-mer counts from the PacBio HiFi reads using meryl (https://github.com/marbl/meryl). The k-mer counts were then used in GenomeScope2.0 (Ranallo-Benavidez et al. 2020) to estimate genome features including genome size, heterozygosity, and repeat content. For contiguity metrics, we ran QUAST (Gurevich et al. 2013). To evaluate genome quality and functional completeness, we used BUSCO (Manni et al. 2021) with the Embryophyta ortholog database (embryophyta_odb10), containing 1614 genes. Assessment of base level accuracy (QV) and k-mer completeness was performed using the previously generated meryl database and merqury (Rhie et al. 2020). We further estimated genome assembly accuracy via BUSCO gene set frameshift analysis using the pipeline described in Korlach et al. (2017). Measurement of the size of the phased blocks is based on the size of the contigs generated by HiFiasm on HiC mode. We follow the quality metric nomenclature established by Rhie et al. (2021), with the genome quality code x.y.P.Q.C, where, x = log10[contig NG50]; y = log10[scaffold NG50]; P = log10 [phased block NG50]; Q = Phred base accuracy QV (quality value); C = % genome represented by the first ‘n’ scaffolds, following a karyotype of 2n = 12, known for the number of chromosomes for this species (Koshy and Mathew 1988; Safonova 1988). Quality metrics for the notation were calculated for the primary assembly.

### Chloroplast genome assembly

The chloroplast sequence for *E. californica* was generated with Oatk (Zhou et al. 2025). We used GeSeq (Tillich et al. 2017) to generate a draft genome annotation. We used the chloroplast genome assembly from *Arabidopsis thaliana* (NCBI:NC000932.1; Sato et al. 1999) to visually validate the order of the sequences that represent the chloroplast regions (Large Single Copy—LSC, Small Single Copy—SSC, Inverted Repeats—IRs). We aligned the generated sequence against the guide using lastz (Harris 2017) and visualized the alignment using LAJ (Wilson et al. 2001). The resulting assembly was annotated using the online version of GeSeq (Tillich et al. 2017) and visualized using the online version of OGDRAW (Greiner et al. 2019). After completing the nuclear genome, we searched for matches of the resulting chloroplast assembly sequence in the nuclear genome assembly using BLAST+ (Camacho et al. 2009). We filtered out contigs and scaffolds from the nuclear genome with a percentage of sequence identity > 99% and size smaller than the chloroplast assembly sequence.

### Comparison to previous E. californica genome assemblies

We compared this genome with another published *E. californica* genome from Hori et al. (2018) from the National Center for Biotechnology using datasets (O’leary et al. 2024, NCBI: GCA_002897215.1). We calculated contiguity metrics using QUAST (Gurevich et al. 2013) and BUSCO (Manni et al. 2021) with the Embryophyta gene set, as we did with the quality assessment of our assemblies.

## Results

### Sequencing data

**Fig. 2:**
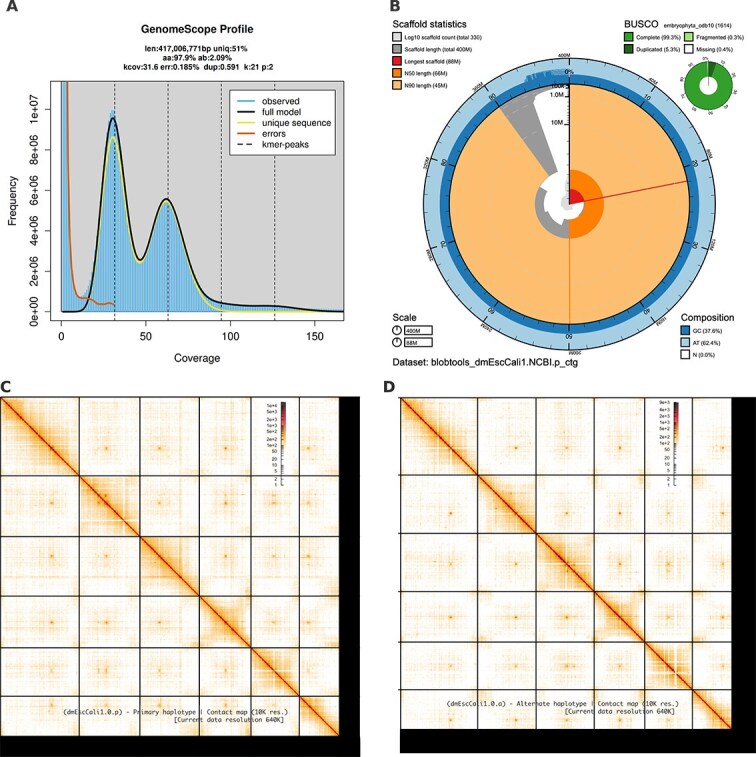
(A) The K-mer spectrum, generated from PacBio HiFi sequencing data using GenomeScope 2.0, shows a clear bimodal distribution. This reflects the diploid nature of *Eschscholzia californica*, with the lower peak representing sequence differences between haplotypes and the higher peak showing shared regions of similarity. (B) the BlobToolKit snail plot gives a breakdown of genome assembly metrics. The first line highlights the longest scaffold, with scaffolds arranged clockwise by size. Arcs show the scaffold N50 and N90 values. The spiral at the center tracks the cumulative scaffold count, with lines marking orders of magnitude. Around this, bands display the GC and AT content distribution. Shaded regions highlight the proportion of undefined bases (Ns) in the assembly. (C-D) Omni-C contact maps for the primary (C) and alternate (D) genome assemblies show how genomic regions are arranged and connected. Each cell represents sequencing data linking two regions, with darker areas showing stronger connections. Scaffolds are separated by lines, making it easy to spot regions with higher or lower fragmentation.

The Omni-C library generated 75.27 million read pairs, and the PacBio SMRTBell library generated 2.33 million HiFi reads. The PacBio HiFi reads yielded ~65X genome coverage and had an N50 read length of 13 666 bp; a minimum read length of 109 bp; a mean read length of 11 723 bp; and a maximum read length of 52 610 bp (see Supplementary Fig. S1 for read length distribution). Based on the PacBio HiFi data, Genomescope 2.0 estimated a genome size of 417 Mb, a 2.09% heterozygosity rate and a 0.185% sequencing error rate. The k-mer spectrum shows a bimodal distribution with a major peak at ~ 31-fold coverage and a minor peak at ~ 60-fold coverage (Fig. 2A).

### **Nuclear** genome assembly

The final genome assembly (dmEscCali1) consists of two phased haplotypes; however, based on our assessment of completeness and contiguity, the assemblies have been tagged as primary and alternate. Both assemblies are similar in size, with a difference of ~ 4.5 Mbs. The final haplotype assemblies are also similar in size but not equal to the estimated genome assembly size from GenomeScope2.0, as observed in other taxa (see Pflug et al. 2020, for example).

The primary haplotype (dmEscCali1.0.p) consists of 331 scaffolds spanning 401.68 Mb with a contig N50 of 11.81 Mp, a scaffold N50 of 66.43 Mb, the largest contig size of 33.92 Mb, and the largest scaffold size of 88.06 Mb. The alternate haplotype (dmEscCali1.0.a) consists of 736 scaffolds spanning 406.19 Mb with a contig N50 of 15.15 Mb, a scaffold N50 of 66.07 Mb, the largest contig size of 36.53 Mb, and the largest scaffold size of 87.74 Mb (Fig. 2B).

The primary haplotype has a BUSCO completeness score for the Embryophyta gene set of 99.2%, a base pair quality value (QV) of 62.33, a k-mer completeness of 74.52%, and a frameshift indel QV of 50.69. The alternate haplotype has a BUSCO completeness score for the same gene set of 99%, a base pair QV of 59.09, a k-mer completeness of 74.12%, and a frameshift indel QV of 50.17.

During manual curation, we made 50 joins (21 on the primary haplotype and 29 on the alternate haplotype) and 7 breaks (2 on the primary haplotype and 5 on the alternate haplotype) based on the signal from the Omni-C contact maps. We filtered out 675 contigs corresponding to chloroplast contaminations (361 from the primary haplotype and 314 contigs from the alternate haplotype). No other contigs were removed.

The Omni-C contact maps show highly contiguous assemblies, with six major scaffolds along the diagonal axis corresponding to the haploid chromosome set (*n* = 6) (Fig. 2C and D). Assembly statistics are reported in Table 2 and represented graphically in Fig. 2C. We have deposited the genome assembly on NCBI GenBank (See Table 2 and Data Availability for details).

**Table 2:** Sequencing and assembly statistics, and accession numbers.

**Bio Projects** **& Vouchers**	CCGP NCBI BioProject			PRJNA720569			
	Genera NCBI BioProject			PRJNA765821			
	Species NCBI BioProject			PRJNA777172			
	NCBI BioSample			SAMN40725789, SAMN40725790			
	Specimen identification			CCGP_15_EC_CP2			
	NCBI Genome accessions			**Primary haplotype**		**Alternate haplotype**	
	Assembly accession			JBCEGH000000000		JBCEGI000000000	
	Genome sequences			GCA_041429995.1		GCA_041429965.1	
**Genome Sequence**	PacBio HiFi reads		Run	1 PACBIO_SMRT (Sequel IIe) run: 2.3 M spots, 27.4G bases, 16.5Gbytes			
			Accession	SRR30832750			
	Omni-C Illumina reads		Run	2 ILLUMINA (Illumina NovaSeq X) runs: 75.2 M spots, 122.7G bases, 7.5Gbytes			
			Accession	SRR30832748,SRR30832749			
	**Genome Assembly Quality Metrics**	Assembly identifier (Quality code^*^)			dmEscCali1(7.7.P7.Q60.C94)		
	HiFi Read coverage^§^			65.65X			
				**Primary haplotype**		**Alternate haplotype**	
	Number of contigs			375		773	
	Contig N50 (bp)			11 819 996		15 159 196	
	Contig NG50^§^			11 819 996		14 141 982	
	Longest Contigs			33 922 680		36 533 502	
	Number of scaffolds			331		736	
	Scaffold N50			66 439 541		66 076 575	
	Scaffold NG50^§^			66 439 541		66 076 575	
	Largest scaffold			88 064 882		87 746 949	
	Size of final assembly			401 694 956		406 202 045	
	Phased block NG50^§^			11 918 934		11 352 675	
	Gaps per Gbp (# Gaps)			110(44)		91(37)	
	Indel QV (Frame shift)			50.69557105		50.17997367	
	Base pair QV			62.3369		59.0983	
				Full assembly = 60.4139			
	k-mer completeness			74.5278		74.1228	
				Full assembly = 99.0587			
	BUSCO completeness (embryophyta) *n* = 1614		C^**^	S^**^	D^**^	F^**^	M^**^
		P^‡^	99.20%	93.90%	5.30%	0.30%	0.50%
		A^‡^	99.00%	93.70%	5.30%	0.60%	0.40%

^*^Assembly quality code x.y.P.Q.C derived notation, from (Rhie et al. 2021). x = log10[contig NG50]; y = log10[scaffold NG50]; P = log10 [phased block NG50]; Q = Phred base accuracy QV (Quality value); C = % genome represented by the first ‘n’ scaffolds, following a karyotype of 2n = 12, known for the number of chromosomes for this species (Genome on a Tree—GoaT; tax_tree (*Eschscholzia californica*); Challis et al. 2023). Quality code for all the assembly denoted by primary assembly (dmEscCali1.0.p) ^‡^(P)rimary haplotype and (A)lternate haplotype assembly values. ^§^Read coverage and NGx statistics have been calculated based on the estimated genome size of 417 Mb. ^**^ BUSCO scores: (C)omplete and (S)ingle; (C)omplete and (D)uplicated; (F)ragmented and (M)issing BUSCO genes. n, number of BUSCO genes in the set/database.

### Chloroplast genome assembly

The final chloroplast assembly of *E. californica* is 160 737 bp long, with 38.7% GC content. The LSC region is 89 850 bp long, the SSC is 19 173 bp long, and the IR is 25 852 bp long. This plastome contains 295 genes, including rRNAs and tRNAs (not counting the duplication of the IR).

## Discussion

This *E. californica* genome provides a high-quality reference to facilitate myriad studies and analyses, including genome evolution, comparative genomics, adaptive genomics, and landscape genomics research. This genome assembly enables ongoing exploration of how *E. californica* adapts to various environments and how its genetic diversity shapes its evolution. With increasing climate variability and environmental stressors projected for California and western North America, having access to a high-quality genome assembly will support investigations into the genetic basis of local adaptation and resilience. The new reference genome serves as a valuable resource for studying the unique traits and genetic variations of *E. californica* within various contexts, including ecological, evolutionary, horticultural, restoration, and conservation studies. More broadly, the genome may also be useful for comparative work across the Papaveraceae, contributing to studies on trait evolution, genome structure, and developmental biology in non-core eudicots.

The improved contiguity, reduced fragmentation, and enhanced resolution of repetitive regions make this genome assembly an improved genomic resource for functional, comparative, and population genomic studies. Hori et al. (2018) developed a reference genome that covers 0.489 Gb (about 97% of the genome); draft genome sequences are available in the DDBJ/GenBank/EMBL databases (accession Nos. BEHA01000001–BEHA01053253; 53 253 entries), and annotated gene information is available at the Eschscholzia Genome Database (http://eschscholzia.kazusa.or.jp). This reference has been used in studies of cytochrome P450 gene diversification and alkaloid biosynthesis, and has contributed to broader comparative analyses of plant genome evolution (e.g., Bowles et al. 2020). This genome has a scaffold N50 of 753 Kb and a contig N50 of 20.6 Kb. The updated assembly improves upon these values by roughly two orders of magnitude, with a scaffold N50 of 66.4 Mb and a contig N50 of 11.8 Mb, and up to ~ 33 Mb of gaps (Supplementary Table 1). The smaller overall assembly likely reflects better resolution of duplicated and repetitive sequences, resulting in a more accurate genome with fewer assembly artifacts (Simão et al. 2015).

The complete genome of *E. californica* enables the future integration of diverse phenotype and population genetics methodologies, allowing researchers to investigate the genetic basis of adaptive traits, such as drought tolerance. An increase in aridity is expected across terrestrial regions globally in the coming decades, even in areas with predicted precipitation increases, due to heightened evaporative demand driven by rising temperatures (Scheff and Frierson 2015; Berg et al. 2016). These shifts raise important questions about how species like *E. californica* will respond, especially in areas already facing water stress. This updated genome can be helpful in cases where individual approaches such as genome scans or environmental associations may not pinpoint loci under selection. (Savolainen et al. 2013; Danilevicz et al. 2020). To support this kind of analysis, the *E. californica* reference genome facilitates whole-genome resequencing efforts to refine estimates of genomic diversity, gene flow, and historical as well as contemporary dispersal across populations. This approach can help identify genetic variants linked to drought response and pinpoint regions of elevated diversity that may contribute to future adaptive potential.

Efforts like the CCGP have expanded our understanding of how genetic diversity is structured across California’s ecosystems and taxonomic groups. Including *E. californica* in these efforts enables researchers to map gene–environment interactions at fine scales and assess how local genetic variation contributes to ecological resilience (Rellstab et al. 2015; Shaffer et al. 2022). This knowledge is important for developing conservation strategies aimed at sustaining populations under changing climate conditions. The *E. californica* reference genome also provides a foundation for restoration planning, as this species is commonly utilized in habitat restoration projects. At the same time, *E. californica* also presents an ecological challenge due to its invasive tendencies in regions where it has been introduced (Pauchard et al. 2016). Understanding the genomic basis of both adaptation and invasiveness can guide when and where to use *E. californica* in restoration, helping balance its benefits with its risks under changing climate conditions (Barrett 2015; Rius et al. 2015). As with any culturally significant species, it is important that genomic data be used in ways that support, rather than disregard, Indigenous land management and stewardship practices. This balance is especially important as climate pressures reshape where and how species persist.

## Supplementary Material

Supplementary_figure_1_E_californica_esaf058

Supplementary_table_1_E_californica_esaf058

## Data Availability

Data generated for this study are available under NCBI BioProject PRJNA777172. Raw sequencing data for samples CP2 and CP5 (NCBI BioSample SAMN40725789, SAMN40725790) are deposited in the NCBI Short Read Archive (SRA) under SRR30832750 for PacBio HiFi sequencing data, and SRR30832748, SRR30832749 for the Omni-C Illumina sequencing data. GenBank accessions for both primary and alternate assemblies are GCA_041429995.1 and GCA 041429965.1, and for genome sequences JBCEGH000000000 and JBCEGI000000000. The GenBank genome assembly for the chloroplast genome is JBCEGH010000331.1. Assembly scripts and other data for the analyses presented can be found at the following GitHub repository: www.github.com/ccgproject/ccgp_assembly.
