# Supplementary material for: A genome assembly of the California poppy, Eschscholzia californica
Source: J Hered. 2025 Aug 28;117(2):309–17. doi: 10.1093/jhered/esaf058 (PMC13017466; doi:10.1093/jhered/esaf058)
Supplement: Supplementary_figure_1_E_californica_esaf058 [file supplementary_figure_1_e_californica_esaf058.pdf]

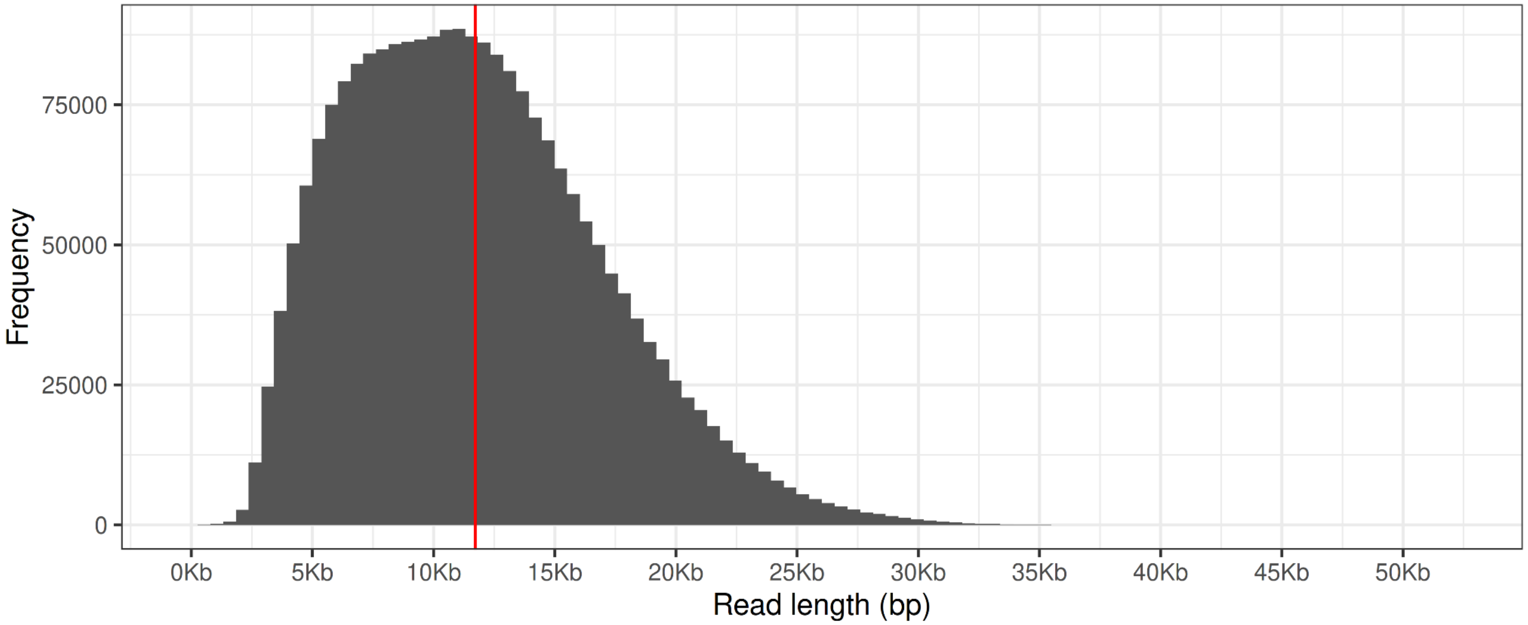

Figure S1. Distribution of PacBio HiFi read lengths. The histogram represents the frequency distribution of PacBio HiFi read lengths (base pairs, bp) obtained from sequencing. The red vertical line indicates the average read length. This figure illustrates the high-quality, long-read sequencing data generated, with the majority of reads concentrated around the average length.
